# Reading the room: Autistic traits, gaze behaviour, and the ability to infer social relationships

**DOI:** 10.1371/journal.pone.0282310

**Published:** 2023-03-01

**Authors:** Leilani Forby, Nicola C. Anderson, Joey T. Cheng, Tom Foulsham, Bradley Karstadt, Jessica Dawson, Farid Pazhoohi, Alan Kingstone

**Affiliations:** 1 Department of Psychology, University of British Columbia, Vancouver, British Columbia, Canada; 2 Department of Psychology, York University, Toronto, Ontario, Canada; 3 Department of Psychology, University of Essex, Colchester, Essex, England; CNRS: Centre National de la Recherche Scientifique, FRANCE

## Abstract

Individuals high in autistic traits can have difficulty understanding verbal and non-verbal cues, and may display atypical gaze behaviour during social interactions. The aim of this study was to examine differences among neurotypical individuals with high and low levels of autistic traits with regard to their gaze behaviour and their ability to assess peers’ social status accurately. Fifty-four university students who completed the 10-item Autism Quotient (AQ-10) were eye-tracked as they watched six 20-second video clips of people (“targets”) involved in a group decision-making task. Simulating natural, everyday social interactions, the video clips included moments of debate, humour, interruptions, and cross talk. Results showed that high-scorers on the AQ-10 (i.e., those with more autistic traits) did not differ from the low-scorers in either gaze behaviour or assessing the targets’ relative social status. The results based on this neurotypical group of participants suggest that the ability of individuals high in autistic traits to read social cues may be preserved in certain tasks crucial to navigating day-to-day social relationships. These findings are discussed in terms of their implications for theory of mind, weak central coherence, and social motivation theories of autism.

## Introduction

Autism is a neurodevelopmental condition characterized by difficulties in social interaction, intense and focused interests, and repetitive behaviours [1], and affects 1 in 66 children in Canada [2]. While some autistic individuals display average to above-average intelligence, have the ability to speak, write, read, and can perform daily self-care rituals (e.g., bathing, eating, etc.), they can still experience difficulties in initiating and ending social interactions, making eye contact, respecting personal space, engaging in the typical back-and-forth rhythm of conversation, regular speech prosody, and forming and maintaining meaningful friendships [3–6]. Aside from those who have a formal autism diagnosis, there is growing evidence that autistic traits exist in the general population [7, 8]. Although originally applied to parents and siblings of diagnosed individuals [9, 10], the term “broad autism phenotype” (BAP) is now used to describe individuals with subclinical levels of autism [11–13], but who nonetheless experience difficulty with social communication, intense preferred interests, or developing friendships or close relationships [12–15]. Relative to those without such traits, individuals with BAP traits are also significantly less likely to attain managerial or professional positions [16], and more likely to experience anxiety or depression [16–18]. Similar to autism, there is evidence that in the BAP, as autistic traits increase, so do difficulties in the social domain [12, 18–20]. In general, research shows that individuals with the BAP can experience autism-related difficulties in social communication [7, 8, 12, 21–24], as well as characteristics such as strong interests and repetitive behaviors [13, 23]. Thus, individuals with autistic traits who do not meet criteria for a diagnosis may still find themselves impacted by autism’s hallmark characteristics. As such, there have been calls for increased investigation into the BAP [7, 18, 25].

The broad impact of these social difficulties raises the question, how do autistic trait levels affect an individual’s ability to “read the room”? Before exploring this issue, we provide a brief review of the three models of autism which will be tested in the current study, as well as theories regarding social hierarchy in humans.

Acknowledging the Canadian Autism Spectrum Disorder Alliance and identity first considerations, this paper will refer to individuals with autism as “autistic individuals”, and autism spectrum disorder as “autism” [26]. Note, however, that the individuals we study here are members of the general population who vary in autistic traits, and to our knowledge none have been diagnosed with autism.

### Autism models

Due to the fact that autism is a multi-faceted condition, with affected individuals exhibiting various difficulties and to varying degrees, many models of autism have been developed. For example, models centered on difficulties in theory of mind (ToM) posit that the social challenges experienced by autistic individuals are rooted in their limited ability to recognize mental states (e.g., beliefs, desires, thoughts, and intentions) in themselves and/or others [8, 27]. However, there is yet to be one clear model for autism stemming from ToM. This is due in part to the fact that ToM difficulties cannot account for all characteristics of autism, and to several studies showing that some autistic individuals pass various ToM tests [28–31]. Despite conflicting results from ToM testing, the extant research supports difficulties with knowing mental states in the self and in others as a core component of autism. Recently, difficulties with ToM have also been found in individuals with the BAP [24, 32, 33].

Another model for autism is the weak central coherence theory (WCC) [34], which posits that autistic individuals do not integrate pieces of information into an organized whole, unlike neurotypicals (NTs) who can do so readily. Past research suggests WCC may also impact individuals with BAP traits [11, 19]. WCC can, for example, account for why autistic individuals have difficulty understanding the subtleties of language. WCC argues that the failure to connect context with meaning is part of the reason why autistic individuals have a difficult time understanding the subtler aspects of communication such as “reading between the lines”. While early WCC described autism as a cognitive dysfunction characterized by a weakness in global processing, it has since evolved to recognize that autism is a cognitive style characterized by superior local processing [31]. The current model also proposes that when given explicit instructions, autistic individuals can process globally to find whole meaning in pieces of information [34, 35], however, this ability does not always extend to social skills [34, 36]. Recent work has also reported that individuals with BAP do not apply contextual information as readily as NT when attempting to recognize facial emotions [11].

A third model, social motivation theory (SMT) [37], proposes that the diminished experience of reward during social interactions causes autistic individuals to not attend to socially salient stimuli. Autistic individuals have different reward processing compared to NTs [38, 39], particularly in the case of social interactions [40–42]. There is evidence to suggest that, relative to NTs, autistic individuals have different amygdala responses to faces and eye contact [41, 43, 44], and that such responses result in reduced association between social interaction and positive reward [45]. Research also suggests that another contributing factor is oxytocin: autistic individuals have been found to have lower blood levels of oxytocin [46], and lower oxytocin levels were associated with reduced sensitivity to social cues [47]. Similarly, individuals high in autistic traits have been found to have increased social anhedonia [48, 49], reduced fixations to socially relevant stimuli [50, 51], and atypical social reward processing relative to NT [42]. Additionally, administration of oxytocin nasal sprays to individuals high in autistic traits has been found to improve their facial emotion recognition [52] and, relative to NT, atypical event-related potential responses to emotional faces [53].

SMT posits that the combined result of the above differences (i.e., reward processing, attention to socially salient stimuli, oxytocin) is low motivation to interact with others. This in turn leads to difficulties in social orienting, seeking and enjoying friendships, and engaging in behaviours that maintain social relationships, all of which have cascading effects on social development. Under SMT, autistic individuals might have the ability to recognize social cues, but do not prioritize them, are not spontaneously motivated by them, and do not recognize their usefulness in reaching relevant goals (e.g., understanding others’ thoughts, reactions, or intentions, predicting behaviour, fostering friendships, etc.). Indeed, previous research has shown that when social cues prove to be a useful tool for completing a task and thus motivation is high, autistic children will attend to those cues in a similar fashion to NT children [54].

In the current study, we draw on the central tenets of these three models—ToM, WCC, and SMT—to guide our inquiry into the potential effects of autistic traits on the processing of social situations.

### Autistic traits and navigating social relationships

The challenges in detecting the thoughts and emotions of others put autistic and high autistic trait individuals at a disadvantage when it comes to responding to different social situations. Autistic individuals have been shown to score lower than NTs in measures that assess ToM [27], cf. [29–31, 55, 56], while both autistic and high trait individuals have been shown to have difficulty reading emotions [20, 57, 58]. Additionally, they may simultaneously broadcast inaccurate or misleading signals of their own. Autistic individuals and those with the BAP can have intense topics of interest [1, 12], and can let these topics dominate their conversations with others [1, 59, 60]. This enthusiasm for a preferred topic may come across as arrogant, or condescending towards others attempting to present their opinions [61–63], and it can be perceived by NTs as bragging about their particular knowledge [61]. Additionally, they tend to disengage when non-preferred topics are being discussed, and thus may appear to be self-centered, disinterested in others, or unable to demonstrate humility or show respect to others.

Overall, these difficulties may pose barriers to building harmonious relationships for individuals with autistic traits (formally diagnosed or exhibiting the BAP). Outside of their own interpersonal relationships, however, there is also reason to surmise that individuals with autistic traits may have difficulties in decoding and inferring the social dynamics between other individuals, a topic that we turn to below.

### Social hierarchy and the ability of individuals with autistic traits to infer status relations

According to the Dominance-Prestige account [64], a prevailing theory of social status, humans possess two independent yet co-existing pathways for gaining social status: prestige and dominance. Under the dominance pathway, high status results from forceful tactics, such as aggressive or intimidating behaviour. These tactics are typically adopted to accumulate and control resources through the induction of fear (for review see [65–67]). Accordingly, subordinates succumb to and monitor the aggressor in order to avoid being on the receiving end of intimidating or violent behaviour, and to safeguard what resources they might currently possess. In short, status is taken and preserved by the domineering individual [68].

How might autistic traits affect one’s ability to infer and recognize dominance-submission asymmetries? Given that individuals with autistic traits have greater difficulty in reading social cues, they may be far less likely to recognize that a message is being sent, which messages are meant for whom, what is being implied, and perhaps most detrimental of all, the signs of imminent danger. Moreover, a person who does not appear to react with fear to cues of intimidation might also be seen as undermining the dominant-styled individual’s authority, and could therefore unknowingly invite additional and escalated dominant tactics. For these reasons, individuals high in autistic traits may experience challenges in the presence of dominant individuals.

In the second avenue to status, prestige, high status is achieved by displaying valuable knowledge, expertise, or skills [64], or for directing their efforts towards maximizing the overall well-being of the group [69]. In this model, lower status individuals track the success of the respected individuals in order to obtain knowledge or learn skills. Leadership through shared knowledge enhances the feelings of fairness in the group, which in turn minimizes conflict. It also tends to coordinate group members’ skills or knowledge for maximum effect. In prestige-based relations, individuals confer high status upon knowledgeable and skilled group members, who have incentives to display prosociality to further augment their status and group-wide cooperation [69]. In such a setting, displays of humility and respect for others are key components for being respected within the group [70], as is working with the group to enhance overall well-being. Individuals high in autistic traits may not recognize when or why others are being deferred to, the interactions that promote group-wide cooperation, and what behaviours might disrupt group goals or harmony, all of which could lead to their own falling in social standing within the group.

### The current study

Through the lens of the Dominance-Prestige theory of social hierarchy, the current study examines the ability of individuals with varying levels of autistic traits to perceive existing status hierarchies as observers. Understanding what college-aged individuals with autistic traits can glean from social interactions could inform career counselling for those with social skills difficulties, and would be timely and beneficial for a group whose next phase in life is entering the workforce.

Here, guided by the aforementioned reasoning, we examine whether individuals with autistic traits may have an attenuated ability to accurately infer relative dominance and prestige relationships in a group context. The current study examines the “reading the room” skills of university students who vary on autistic traits, and is modeled after studies reported in Foulsham et al. [71] and Cheng et al. [65]. In Cheng et al. [65], individuals were filmed while engaging in a group task, after which they were asked to rate fellow group members on their social influence, prestige, and dominance. A second group of participants then viewed the video recorded clips of the original group, and rated the onscreen individuals on the same domains. Cheng et al. [65] found that the status, prestige, and dominance ratings from the original group members converged with the ratings supplied by the participants who watched their interactions via video. This demonstrated that outside observers can accurately assess the status, prestige, and dominance of others, even when those others are only on a subset of video footage. In Foulsham et al. [71], participants viewed the same video tape interactions as in Cheng et al. [65], and revealed that participants’ fixation count and dwell times on the high status people in the videos were greater than for that on the medium status individuals, who in turn were fixated on more frequently and for longer periods of time than the low status individuals.

The present research has two primary goals. First, we recruited participants with varying levels of autistic traits to determine if they differ in their ability to interpret verbal and social cues (such as speech content, as well as facial expressions, tone of voice, and eye contact) as an outside observer, and to accordingly assess the status conferred to people by their fellow group members. This extends prior work such as that reported in Foulsham et al. [71] and Cheng et al. [65].

Our participants were recruited from a university population and their autistic trait levels were measured by the 10-item Autism quotient (AQ-10) [72]. We did not screen for clinical levels of autism and, as such, we could not determine whether or not any of our participants had a formal diagnosis of autism. However, the AQ-10 has been used in previous research to identify high trait individuals [73–76], and because we were interested in the effects of autistic traits on “reading the room” skills, the AQ-10 was used to identify the high and low trait individuals among our participants. Given that autistic individuals and those with BAP traits may have difficulty decoding social cues [1, 24, 60], we predict that relative to people low in autistic traits, individuals high in autistic traits will be compromised in their ability to discriminate between prestige-styled or dominance-styled tactics employed to attain status, and that their assessment of the relative status conferred to people by their fellow group members will differ from the assessment made by individuals low in autistic traits.

All three models of autism, theory of mind (ToM), weak central coherence (WCC), and social motivation theory (SMT), support these predictions. According to ToM, high autistic trait individuals will not be able to infer the intentions behind tactics meant to either intimidate or persuade fellow group members. Additionally, they should not be able to deduce the group status, prestige, and dominance conferred onto an individual because they have limited “I think she thinks” abilities. According to WCC, individuals with autistic traits will have difficulty extrapolating a person’s social standing because they will not piece together relevant information (e.g., getting talked over or cut-off, group members laughing at their jokes, etc.), and measure such information against the context of all six video clips. In terms of SMT, even if individuals with autistic traits are capable of recognizing social cues, they might not be motivated to attend to these cues, or prioritize them in a manner that helps deduce a person’s group standing.

Second, the current study will test whether individuals high in autistic traits differ from low trait individuals in the way that they observe dynamic social interactions, vis-a-vis their eye movement behaviour. Because the extant research in this area is mixed for adolescents and adults [77–79], cf. [80], we make no firm predictions regarding whether or not young adults high in autistic traits will behave differently from low trait individuals in the way that they visually monitor the social interactions. However, it follows that if individuals high in autistic traits rate people in a video differently than low trait individuals on social status, prestige, and/or dominance, then those higher in autistic traits may also look at those individuals differently. Thus, we assessed how individuals are looked at with regard to their body, head, and eyes. Indeed, looks to the eye regions of those in the videos are of particular interest. Although research shows that autistic children make less eye contact [45, 81, 82], results become mixed as the age of participants increases [44, 83, 84]. Nevertheless, the general weight of empirical evidence supports the prediction that high autistic trait individuals will look less to the eyes than low autistic trait individuals do.

## Methods

### Participants

Using the Fabs package [85] in RStudio [86], it was determined that 41 participants were needed to achieve a power of .80 at a significance level of .05. The nature of recruiting from the Human Subject Pool at the University of British Columbia resulted in our exceeding this target, with 64 participants taking part in exchange for extra credit. The study was approved by the university’s Behavioural Research Ethics Board.

Participants’ individual 10-item Autism Quotient (AQ-10) [72] scores, and the mean AQ-10 score (*M* = 4.94, *SD* = 2.57) for all responses to the survey, were calculated. No participants received an AQ-10 score of 0 or 10. Results from 10 individuals were not used in the analysis because they did not complete the AQ-10, or because their eyes could not be calibrated properly, leaving 54 participants in total. As an individual who scores 6 or higher on the AQ-10 is referred for a full diagnostic assessment [72], this became our high scorers group (HAQ). Twenty-five (16 women, 8 men, 1 other) of our participants met this cut-off with scores of either 6 (*n* = 1), 7 (*n* = 14), 8 (*n* = 7), or 9 (*n* = 3). Twenty-nine (21 women, 8 men) had low scores (LAQ) of either 1 (*n* = 5), and 2 (*n* = 7), 3 (*n* = 8), 4 (*n* = 8), or 5 (*n* = 1). In the absence of a diagnosis, however, we take a conservative position and assume that our study is composed of two groups of neurotypical participants with “high traits” and “low traits”. Note that although our participants were not screened for autism, the AQ-10 scores for this group of high-scorers (N = 25; 46%) met the cut-off for a referral for a full diagnostic assessment.

### Stimuli

The stimuli were the same as the videos used by Foulsham et al. [71] and Cheng et al. [65]. These videos were created using unconcealed video cameras while six unacquainted undergraduates completed a group decision-making task called the Lost on the Moon task [87]. Each group was given a list of 15 items (e.g., signal flare, oxygen tank, pistol), and asked, “Which items would your group need to survive if marooned on the moon?” The groups were then seated around a table (three participants on each side), and given 20 minutes to discuss and rank the items. To incentivize active engagement, participants were told a monetary bonus would be given to the group whose rankings were closest to the correct answer.

**Fig 1:**
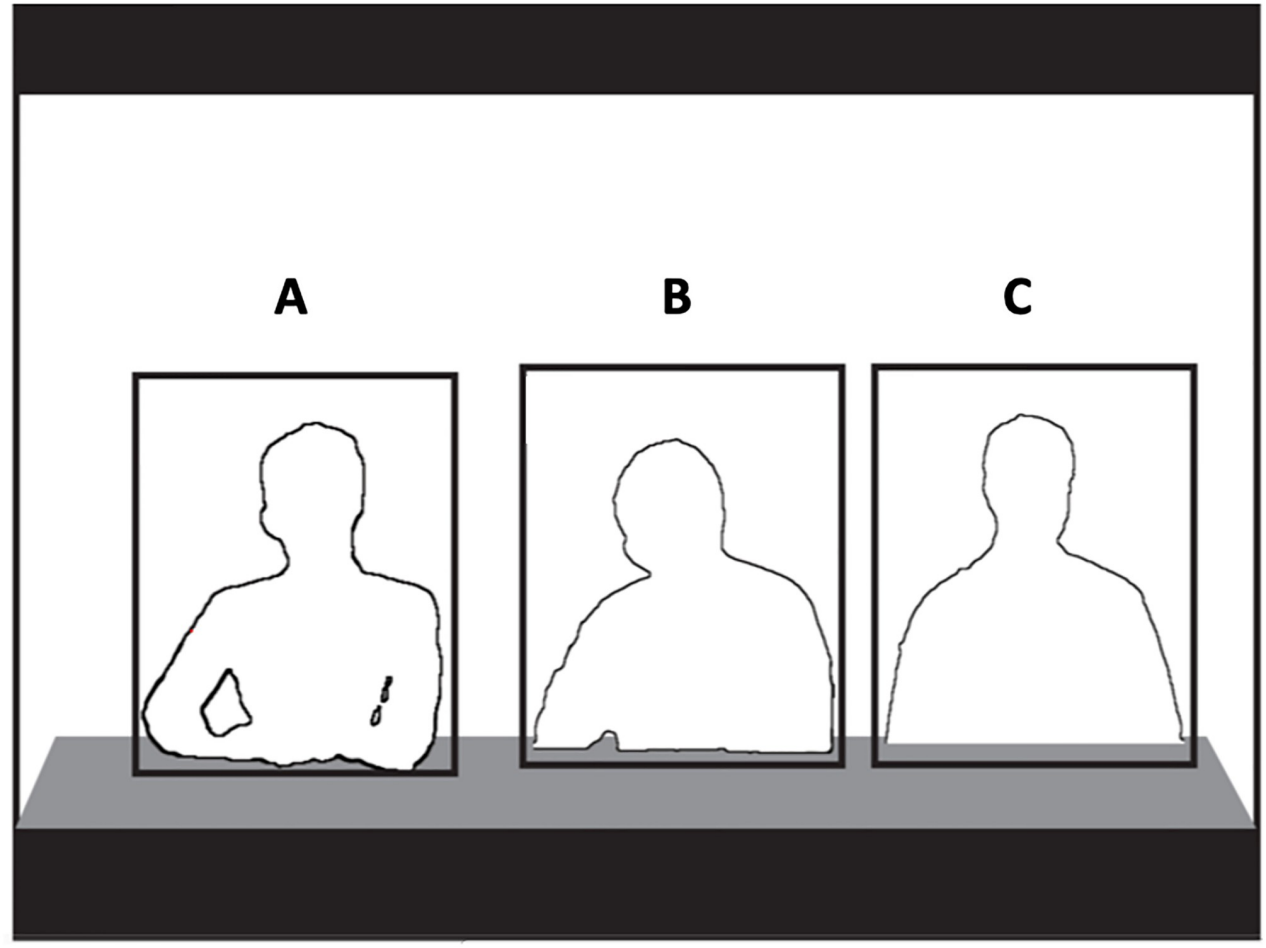
Participants’ view of targets in the video clips. *Note*. Example of the view of targets presented to participants (only the schematic is presented here to protect the privacy of the targets). After viewing the clips, and before completing the Dominance and Prestige Peer Rating Scales [88] for each target, participants were given a screenshot from the videos that identified the targets as A, B, or C. Adapted from “Gaze Allocation in a Dynamic Situation: Effects of Social Status and Speaking,” by Foulsham, et al., 2010. *Cognition*, *117*(3), p. 319–331.

There were four sets of videos, each containing six 20-second clips featuring three participants (“targets”) in the group sitting on one side of the table (Fig 1). These video clips were selected because they captured moments of debate, negotiation, persuasion, or conflict that led to a group decision. In short, the clips featured instances where tactics of dominance or prestige were most actively utilized.

The videos were digital film files with dimensions of 1024 by 768 pixels, and a frame rate of 30 fps. Because the aspect ratio of the original film was 16:9, the clips were presented onscreen with a black border above and below their image. Sound was played through wired headphones.

The choice of video set for each participant was randomized. To increase the difficulty of this status-inference task, the order of the 6 video clips watched was also randomized (i.e., not in chronological order) for a given participant.

### Measures

#### Autistic traits

All participants completed the short AQ-10 [72] prior to the experiment. The AQ-10 is a self-report measure that includes 10 items from the 50-item Autism Quotient (AQ) [8], which is used for adults with typical intellectual functioning. The AQ-10 was designed for clinicians who needed a less time-consuming screening method for autism, and has been shown to perform comparably to the full AQ [89]. Allison et al. [72] found the AQ-10 scored reliably in sensitivity (0.88), specificity (0.91), and positive predictive value (0.85), and yielded mean scores of 7.93 in autistic individuals, and 2.77 in NTs. Additionally, the AQ-10 has been used to identify people high in autistic traits in previous studies [73–76].

#### Assessments of status relations

Using a 7-point Likert scale ranging from 1- not at all, to 7- very much, participants were asked to rate each target using the Dominance and Prestige Peer Rating Scales [88]. The key elements are described below.

*Dominance vs prestige*. Seven items are used to measure Dominance (e.g., “He/she often tries to get his/her own way regardless of what others in the group may want”), and 8 items are used to measure Prestige (e.g., “His/her unique talents and abilities are recognized by others in the group”). Target scores for all seven Dominance items are combined to calculate a target’s composite Dominance score, as evaluated by each individual participant. A separate composite Prestige score was calculated from the eight Prestige items as rated by each participant.

*Perceived influence*. Participants also rate targets on the target’s ability to influence the group with three items: “led the task”, “was paid attention”, and “had high status”. Scores for these items are combined to calculate a target’s overall perceived status within their group.

### Equipment

Stimuli were controlled by the SensoMotoric Instruments (SMI) Experiment Center 3.4 and displayed on the SMI-issued 22” Dell color monitor. Eye tracking was done with the SMI iViewX 2.8 Red to synchronize data collection with the videos being played through Experiment Center. Pupil data was taken from the left eye at 500 Hz, and fixations were defined using velocity (30°/s) and acceleration thresholds (8000°/s). While the SMI equipment does not use a head rest to standardize participants’ distance from the screen, its program provides clear, onscreen cueing to ensure participants are seated within a target range to the sensors. The average distance from the screen for participants was 53 cm. The DPRRS was completed on a Dell Latitude E6530 laptop with a 15.6” display.

### Procedure

All participants provided written consent prior to the start of the experiment. Immediately following that, the experiment began with on-screen instructions that explained the format of the study (e.g., a series of videos will be followed by questionnaires). Per the original studies, participants were instructed to watch the videos and “think about who you would want to work with on a subsequent task.” This directive was part of the instructions given to observers of the video clips in order to maximize the feeling of being in the room with the targets. Before each clip was shown, a 5-point eye calibration was performed. The volume was adjusted according to participant preference.

After watching all six clips, participants then completed the DPRRS on the laptop. As a memory prompt for the questionnaires, participants were given a printed screen shot of the targets they had watched, with each target identified as “A”, “B”, or “C”. The items within each questionnaire were randomized, as was the order of the targets (e.g., some participants answered randomized questions related to target A first, others answered randomized questions related to target C first, etc.).

### Data analysis

The R packages afex [90], rstatix [91], and emmeans [92] were used for analysis and all follow-up tests. Figures were created with ggplot 2 [93]. For all anovas, where violations of sphericity occurred, Greenhouse-Geiser adjustments to the degrees of freedom were made.

Participants rated each person (“target”) in the video on three domains: status, prestige, and dominance. From these ratings, we then calculated the overall score for each domain to determine the relative rank (‘high’, ‘medium’, or ‘low’) of each target. Where a given participant’s overall score for a domain resulted in two targets with the same domain score, the participant’s AQ-10 group (high scorers vs. low scorers on the AQ-10) consensus was used to resolve those ties. As each participant’s AQ-10 score was used for group assignment, we reasoned that it was consistent to apply group membership to resolve ties. Note, however, that use of group assignment to resolve ties was necessary for less than 4% of the ratings.

Using participant domain rankings, fixation data were organized such that if a participant had given a specific target the highest status rating, for example, all fixations to that target were marked as fixations to the “high” status target. The same was done for the low and medium ranked targets. Proportions to each area of interest (AOI) were calculated per participant, per trial. This was done for all participants across all four video batches.

Next analyzed were the fixations to the target in the video clips. An AOI was created for each target. Due to the fact that targets often moved during the video clips, dynamic, rather than fixed, full body AOIs (head, eyes, and torso AOIs combined into one) were created to follow each target’s movements. All full body AOIs in each video clip were measured as a percentage (*M* = 10.2, *SD* = .42) of the total onscreen image, and were not significantly different across all targets in all video clips, *F*(2, 6) = .43. That is, each target’s full body AOI occupied a similar percentage of the onscreen image. A fixation that landed within a full body AOI of a target was assigned to that target. Fixations to the three targets’ full body AOIs in each video clip amounted to 82% of the fixations. Given that the full body AOIs amounted to roughly 30% (*M* = 30.5, *SD* = 2.53) of the total screen image, the data suggest the targets attracted more attention than the background objects.

## Results

There was a significant difference in mean AQ-10 scores between low scorers (LAQ; *M* = 2.71, *SD* = 1.15), and high scorers (HAQ; *M* = 7.35, *SD* = 1.02), *t*(52) = 16, *p* < .001, *d* = 4.26. This clear distinction in autistic trait scores between the two group means is in line with previous AQ-10 research [72, 89], and demonstrates a clear difference between the high and low groups in their autistic trait levels.

### Ratings

Table 1 presents the social influence (status), prestige, and dominance ratings made by HAQ and LAQ. Statistical results for each domain are outlined below.

**Table 1:** LAQ and HAQ participants’ status-relevant ratings of targets.

	Status			Prestige			Dominance		
	Low	Medium	High	Low	Medium	High	Low	Medium	High
Overall									
Mean	2.41	3.94	5.36	3.21	4.46	5.22	1.57	2.66	4.11
*SE*	*0*.*11*	*0*.*11*	*0*.*11*	*0*.*10*	*0*.*10*	*0*.*10*	*0*.*11*	*0*.*11*	*0*.*11*
LAQ									
Mean	2.20	3.83	5.30	2.95	4.33	5.30	1.37	2.57	3.97
*SE*	*0*.*14*	*0*.*14*	*0*.*14*	*0*.*11*	*0*.*13*	*0*.*13*	*0*.*08*	*0*.*17*	*0*.*21*
HAQ									
Mean	2.64	4.06	5.41	3.50	4.60	5.15	1.82	2.78	4.22
*SE*	*0*.*15*	*0*.*17*	*0*.*17*	*0*.*18*	*0*.*13*	*0*.*12*	*0*.*12*	*0*.*17*	*0*.*23*

*Note*. Mean ratings given to targets by participants. For each domain (Status, Prestige, Dominance), targets with the highest relative rating in the group of targets were categorized as “High”, targets with the lowest relative rating were categorized as “Low”, with the remaining target catergorized as “Medium”. SE = Standard error; LAQ = low AQ-10 scorers (i.e., score of 5 or lower on AQ-10); HAQ = high AQ-10 scorers (i.e., score of 6 or higher on AQ-10).

#### Social influence (status)

A mixed model analysis of variance (ANOVA) was conducted, all with Bonferroni-corrected pairwise comparisons, to determine if there was an effect of AQ-10 score on the status scores of the targets, with AQ-10 score (HAQ and LAQ) as a between-group factor, and target ratings (low, medium, and high) as a within-group factor. There was no main effect of AQ-10 score, *F*(1, 46) = 2.86, *p* = .097, $\eta_{p}^{2}=.06$, 90% CI [0.00, 0.20]. Additionally, there was no significant effect observed for the interaction of AQ-10 score and target ratings, *F*(2, 92) < 1. This indicates autistic traits did not affect the ratings of status assigned to the targets.

However, we found a significant difference in the status ratings themselves, *F*(2, 92) = 214.05, *p* < .001, $\eta_{p}^{2}=.82$, 90% CI [0.77, 0.86: the low status target (*M* = 2.41, *SE* = .11), was rated significantly lower in status than the medium status target (*M* = 3.94, *SE* = .11), *t*(92) = 10.77, *p* < .001, which was in turn rated significantly lower than the high status target (*M* = 5.36, *SE* = .11), *t*(92) = 9.92, *p* < .001. Taken together, these data suggest that the HAQ and LAQ groups did not differ significantly in how they discriminated relative social hierarchy.

#### Prestige

Following status, the prestige scores of the targets were analyzed using a similar analysis used for status. There was no significant main effect of AQ-10 score, *F*(1, 46) = 2.58, *p* = .115, $\eta_{p}^{2}=.05$, 90% CI [0.00, 0.19]. However, there was a significant difference in the prestige scores of the targets, *F*(2, 92) = 140.84, *p* < .001, $\eta_{p}^{2}=.05$, 90% CI [0.00, 0.19]: the low prestige target (*M* = 3.21, *SE* = .10) was rated significantly lower than the medium prestige target (*M* = 4.46, *SE* = .10), *t*(92) = 10.32, *p* < .001, which was rated lower than the high prestige target (*M* = 5.22, *SE* = .10), *t*(92) = 6.30, *p* < .001. There was an interaction between AQ-10 score and prestige rank, *F*(2, 92) = 4.61, *p* = .012, $\eta_{p}^{2}=.09$, 90% CI [0.01, 0.19], such that HAQ (*M* = 3.50, *SE* = .18) gave significantly higher scores than LAQ (*M* = 2.95, *SE* = .11), *t*(42) = 2.6, *p* = .01, to the low prestige target. However, there were no significant differences between the two groups in their scores for the medium target (HAQ: *M* = 4.33, *SE* = .13; LAQ: *M* = 4.60, *SE* = .13, *t*(52) = 1.4, *p* = .20), or the high target (HAQ: *M* = 5.15, *SE* = .11; LAQ: *M* = 5.30, *SE* = .11, *t*(52) = 0.87, *p* = .40). Although HAQ gave higher scores than LAQ to the lowest ranking target, these data suggest the two AQ-10 groups agreed on the relative prestige ranks of the targets.

#### Dominance

Similar to both status and prestige, a mixed model ANOVA was conducted to test the effect of AQ10 on ratings of targets’ dominance, and found no main effect of AQ-10 score, *F*(1, 46) = 2.39, *p* = .129, $\eta_{p}^{2}=.05$, 90% CI [0.00, 0.18]. There was, however, a significant difference in target dominance ratings, *F*(2, 92) = 185.32, *p* < .001, $\eta_{p}^{2}=.80$, 90% CI [0.74, 0.84]: Pairwise comparisons showed that the low dominance target (*M* = 1.57, *SE* = .11) was rated significantly lower than the medium dominance target (*M* = 2.66, *SE* = .11), *t*(92) = 8.22, *p* < .001, which in turn was rated significantly lower than the high dominance target (*M* = 4.11, *SE* = .11), *t*(92) = 10.97, *p* < .001. There was, however, no significant interaction between AQ-10 score and target dominance rating, *F*(2,92) < 1. Taken together, the data suggest that the two AQ-10 groups did not differ significantly in how they rated the targets’ relative dominance.

### Do autistic traits predict differences in general viewing patterns?

**Fig 2:**
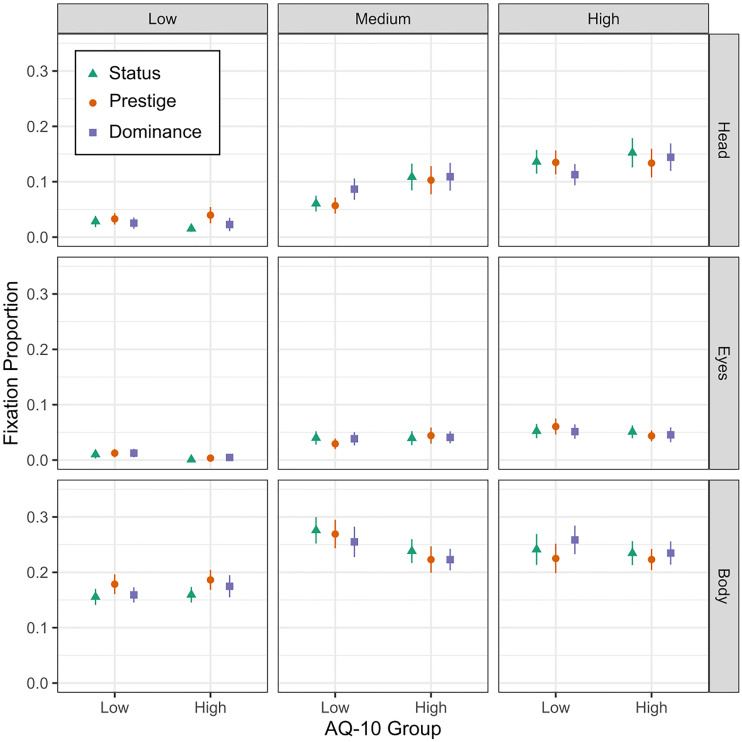
Proportions of fixations to body, head, and eyes. *Note*. Proportions of fixations to the areas of interest (AOIs) for Status (green triangles), Prestige (orange circles), and Dominance (purple squares) by AQ-10 group.

Fig 2 presents the fixation data for the AQ-10 groups as a function of AOI and target status. Fixation results for each domain are outlined below. All background fixations were removed from this analysis, and the resulting proportion data represents the proportion of fixations to each AOI region out of the total number of fixations to the people in the videos, calculated separately for the low and high AQ-10 groups.

The average number of fixations for each 20 second video clip was 46.82 fixations (*SD* = 9.86). A Welch’s t-test was conducted to compare the effect of AQ-10 scores on the number of fixations and found no significant effect, *t*(50) = 0.66, *p* = .500, *d* = .18, between low scorers (*M* = 47.7, *SD* = 9.29) and high scorers (*M* = 45.9, *SD* = 10.54). These results indicate that the high and low AQ scorers (HAQ and LAQ) made a similar number of fixations while viewing the clips.

#### Status

A mixed model ANOVA was then performed, all with Bonferroni-corrected pairwise comparisons unless otherwise noted, to determine the effect of status on fixations to target AOIs, with AQ-10 score (HAQ and LAQ) as a between-group factor, and target status (low, medium, and high) and AOI (body, head, and eyes) as a within-group factors. There was a significant effect of status on fixation proportions, *F*(1.92, 99.73) = 54.03, *p* < .001, $\eta_{p}^{2}=.51$, 90% CI [0.42, 1]: Targets rated as low status (*M* = 0.06, *SE* = .005) had a significantly lower proportion of fixations than the targets rated as medium status individuals (*M* = 0.13, *SE* = .005), *t*(104) = 7.78, *p* < .001, and high status individuals (*M* = 0.14, *SE* = .005), *t*(104) = 9.86, *p* < .001. While high status targets attracted a numerically higher proportion of fixations than medium status targets, the difference between the two ranks was not significant, *t*(104) = 2.08, *p* = .10. Additionally, there was no main effect of AQ-10 score, *F* < 1, and no significant interaction between AQ-10 groups and target status, *F* < 1.

There was also a significant effect of AOI on fixations, *F*(1.67, 86.88) = 344.77, *p* < .001, $\eta_{p}^{2}=.87$, 90% CI [0.84, 1]: The eye AOI (*M* = .03, *SE* = .004) garnered a significantly smaller proportion of looks than did the head (*M* = .08, *SE* = .004), *t*(104) = 7.02, *p* < .001, which in turn garnered a lower proportion of looks than did the body (*M* = .22, *SE* = .004), *t*(104) = 18,40, *p* < .001. This pattern among the AOIs held for the prestige and dominance analyses, as well.

There was no significant interaction between AOI and AQ-10 score, *F*(1.67, 86.88) = 2.30, *p* = .116, $\eta_{p}^{2}=.04$, 90% CI [0.00, 0.11]. Additionally, there was no interaction between AOI and status rank, *F*(1.53, 79.53) = 2.77, *p* = .08, $\eta_{p}^{2}=.05$, 90% CI [0.00, 0.09], and no interaction between AOI, status rank, and AQ-10 score, *F* < 1.

Combining the above results, the data indicate that while the status of an individual can impact the gaze behaviour of spectators, HAQ spectators do not differ significantly from LAQ spectators in how they watch low-, medium-, and high-status actors.

#### Prestige

To examine if prestige had an effect on fixations to targets, the same method for organizing the status fixation data was used for organizing the prestige fixation data (e.g., fixations made to the highest rated target for prestige were considered fixations to the “high prestige” target). Accordingly, the same analysis used to analyze the effect of status was conducted to determine the effect of prestige on fixations, and found a significant effect of prestige on fixation proportions, *F*(1.98, 102.72) = 19.20, *p* < .001, $\eta_{p}^{2}=.27$, 90% CI [0.15, 0.38]: Targets rated as low prestige (*M* = .08, *SE* = .006) received a significantly lower proportion of fixations than those rated as medium prestige (*M* = 0.12, *SE* = .006), *t*(104) = 4.42, *p* < .001, and high prestige (*M* = 0.14, *SE* = .006), *t*(104) = 5.97, *p* < .001. However, there was no significant difference in fixation proportions between those rated as medium and high prestige, *t*(104) = 1.55, *p* = .372. Additionally, there was no main effect of AQ-10 score, *F* < 1, and no significant interaction between AQ-10 scores and target prestige, *F* < 1.

There was no interaction between AOI and AQ-10 score, *F*(1.67, 86.88) = 2.30, *p* = .116, $\eta_{p}^{2}=.04$, CI 90% [0.00, 0.11]. Furthermore, there was no interaction between AOI and prestige rank, *F*(1.98, 103.01) = 1.85, *p* = .163, $\eta_{p}^{2}=.03$, CI 90% [0.00, 0.07], and no interaction between AOI, prestige rank, and AQ-10 score, *F* < 1.

In sum, the prestige results suggest that low prestige targets attract less attention than both medium and high prestige targets, but that there is no significant difference between HAQ and LAQ in how they allocate their attention to low-, medium-, and high-prestige targets.

#### Dominance

Fixation data for dominance was organized the same way status and prestige were (e.g., fixations made to the highest rated target for dominance were considered fixations to the “high dominance” target). These data were analyzed as before, and a significant effect of dominance ranking on fixation proportions was returned, *F*(1.87, 97.11) = 36.62, *p* < .001, $\eta_{p}^{2}=.41$, 90% CI [0.29, 0.51]: The targets rated with low dominance (*M* = .07, *SEM* = 0.005) garnered a significantly lower proportion of fixations than both targets rated with medium dominance (*M* = 0.13, *SEM* = 0.005), *t*(104) = 6.41, *p* < .001, and high dominance (*M* = .14, *SEM* = .005), *t*(104) = 8.12, *p* < .001. There was no significant difference between the targets rated with medium and high dominance, *t*(104) = 1.71, *p* = .269. Additionally, there was no main effect of AQ-10 score, *F* < 1, and no significant interaction between AQ-10 scores and dominance, *F* < 1.

There was no significant interaction between AOI and AQ-10 score, *F*(1.67 86.88) = 2.30, *p* = .116, $\eta_{p}^{2}=.04$, 90% CI [0.00, 0.09], no interaction between AOI and dominance rank, *F*(1.61, 83.85) = 1.18, *p* = .303, $\eta_{p}^{2}=.02$, 90% CI [0.00, 0.02], and no interaction between AOI, AQ-10 score, and dominance rank, *F* < 1.

The overall dominance results show that targets lowest in dominance draw consistently less attention from both AQ-10 groups, but that there is no significant difference between HAQ and LAQ in how they attend to the ranks of dominance.

#### Fixations to eyes

Table 2 presents the fixation data to the eye AOIs for the AQ-10 groups as a function of target domain ratings.

**Table 2:** Fixations to the eyes of low, medium, and high ranked targets.

		Target Rank		
		Low	Medium	High
***Status***				
**Overall**	Mean	0.006	0.040	0.052
	*SE*	*0*.*004*	*0*.*007*	*0*.*008*
**LAQ**	Mean	0.010	0.040	0.052
	*SE*	*0*.*007*	*0*.*012*	*0*.*013*
**HAQ**	Mean	0.001	0.040	0.051
	*SE*	*0*.*000*	*0*.*013*	*0*.*012*
***Prestige***				
**Overall**	Mean	0.008	0.037	0.052
	*SE*	*0*.*004*	*0*.*009*	*0*.*009*
**LAQ**	Mean	0.013	0.029	0.061
	*SE*	*0*.*007*	*0*.*010*	*0*.*014*
**HAQ**	Mean	0.004	0.044	0.044
	*SE*	*0*.*002*	*0*.*015*	*0*.*010*
***Dominance***				
**Overall**	Mean	0.009	0.040	0.049
	*SE*	*0*.*004*	*0*.*008*	*0*.*009*
**LAQ**	Mean	0.013	0.038	0.051
	*SE*	*0*.*008*	*0*.*012*	*0*.*013*
**HAQ**	Mean	0.005	0.041	0.046
	*SE*	*0*.*004*	*0*.*011*	*0*.*013*

*Note*. SE = Standard error; LAQ = low AQ-10 scorers; HAQ = high AQ-10 scorers.

To discern if HAQ (M = 0.031, SEM = 0.005) and LAQ (M = 0.034, SEM = 0.005) looked at the eyes of the targets differently, we conducted a Welch’s t-test between the two groups and found no significant difference: t(160) = 0.41, p < 1.

## Discussion

The current study compared neurotypical individuals with varying levels of autistic traits on two fronts: their ability to assess peers’ social standing within a group, and their gaze behaviour while viewing filmed social interactions of a group task. The video clips of this task completion, which included moments of debate, humor, interruptions, and cross talk, simulated a natural social interaction an individual would likely experience during any given day. It also presented an opportunity to examine the gaze behaviour and subsequent interpretations of these interactions by individuals with high autistic traits, providing insight into how these individuals observe social interactions, and what they glean from them.

The first goal of the current study involved comparing the social observational skills of the AQ-10 groups, low (LAQ; score of ≤ 5), and high (HAQ; score of ≥ 6), to determine if they differed in inferring the relative levels of social influence or status the targets had attained within their groups. We also wanted to determine if HAQ would identify which targets had taken the prestige or dominance path to attain that status. Given that individuals with autistic traits can have difficulty with understanding non-verbal cues, such as body language, facial expressions, tone of voice, and eye contact [1], we predicted that HAQ would differ from LAQ in their assessment of the perceived status of a target within a group, or which tactics (prestige or dominance) had been utilized to attain status.

The results showed however that HAQ did not differ significantly from LAQ in assessing the relative rank of the targets’ status. Also counter to our predictions, HAQ did not differ from LAQ on the prestige and dominance ratings given to the targets, although their mean score for the low prestige target was significantly higher than LAQ’s. Recall that prestige on the DPPRS was probed with questions such as, “His/her unique talents and abilities are recognized by others in the group”, and that dominance was probed with questions such as, “He/she often tries to get his/her own way regardless of what others in the group may want.” The results from the DPPRS suggest HAQ recognized both prestige-styled and dominance-style tactics, and perhaps more importantly, the impact such tactics had on the rest of the group. As HAQ were capable of integrating and translating prestige and dominance information into an overall picture of status, such results are not consistent with weak central coherence theory (WCC) [34, 94] which posits that autistic individuals have difficulties in integrating pieces into a coherent whole. Additionally, HAQ understood the intentions behind a target adopting prestige-style versus dominance-style tactics, as well as the group members’ reactions to these tactics. This is not consistent with theory of mind (ToM) [27], which posits autistic individuals have difficulties in recognizing beliefs, desires, thoughts, and intentions in themselves and others. Our results are somewhat in line with those of Ogawa et al., [95] who found that autistic children performed better than TD children in assessing social hierarchy between two human-like figures in static drawings. However, unlike in Ogawa et al. [95] our adult participants viewed footage of social interactions between live targets (university students). Additionally, participants in the current study were then asked not to assess the targets’ status, but to determine the status afforded to each target *by their peers*, or in other words, to “read the room”. While our HAQ participants did not outperform the LAQ participants as they did in Ogawa et al. [95], the task for our older participants was more demanding: To assess how real humans engaged in conversation were perceived by their conversation partners.

That HAQ performed similarly to LAQ in the current study could be due to the fact that HAQ participants attended to and understood the verbal and non-verbal reactions to the onscreen individuals, although this would be in contrast to much of the literature regarding the social skills of individuals high in autistic traits [6, 60, 61, 96, 97]. Consideration, then, should be given to the design of the study, itself: “Think about who you would like to work with on a subsequent task”. This directive, given before watching the video clips, provided a specific reason to attend to social interactions, which may have aided HAQ. Specifically, whereas LAQ may have attended to the social interactions spontaneously, it is possible that HAQ did so because of the task instruction. This interpretation would dovetail with social motivation theory (SMT) [37] which proposes that when given an explicit instruction, individuals with autistic traits can attend to verbal and non-verbal social cues. The results thus can be reconciled with SMT; though it also begs that the present study be repeated in the future without providing the participants with a viewing instruction.

After establishing that HAQ did not differ significantly from LAQ in their assessment of others’ group member status, the next goal of the current study was to compare the viewing behaviour of LAQ and HAQ to determine if there were differences in gaze behaviour while viewing dynamic social scenes. The overall findings (Fig 2) were that HAQ average fixation proportions to targets’ AOIs were similar to LAQ, and statistical analyses confirmed this. This was also true regardless of the domain (status, prestige, and dominance) or rank (e.g., low, medium, and high) being probed. In other words, HAQ gaze behaviour while watching dynamic social interactions was not significantly different from the gaze behaviour of LAQ.

Regarding fixations to the eyes, the difference between the two groups was not significant. Interestingly, HAQ participants had a numerically higher proportion of looks to the eyes than did LAQ for both the medium dominance and medium prestige targets. Although autistic individuals have been shown to display elevated threat responses to faces and eyes [43, 44, 98, 99], it could be possible that the directive to find “who you would like to work with on a subsequent task”, provided HAQ with a task that could be solved by attending to social cues. Under SMT, this directive created a specific need to ascertain which targets were leading the task due to either having useful knowledge (prestige), or by using intimidating tactics (dominance), and thus provided motivation to override any natural tendency to avoid attending to the eyes. As alluded to previously, the current study’s outcome might have been different had the participants been allowed to freely view the video clips, with no thought to future hypothetical work partners. Future studies could examine if HAQ perform comparably to LAQ when no directive is given before viewing the video clips. In the present study, however, one can confidently conclude that when HAQ individuals are instructed to “read the room”, the manner in which they do so, and what they take away from that assessment, is comparable to LAQ individuals.

One potential limitation to the present study was using the AQ-10 to identify participants high in autistic traits, rather than recruiting individuals formally diagnosed with autism. As such, we caution that our findings should be conservatively inferred as addressing the gaze behaviour of individuals with higher autistic traits, but do not speak directly to the gaze patterns of autistic individuals (who have been clinically diagnosed). However, the AQ-10 has been shown to be comparable to the full-length AQ in identifying individuals with autistic traits [72, 89]. It has also been used in previous studies where identifying autistic trait levels was a key component of the study [73–76, 100, 101]. Thus, the results here suggest HAQ participants were accurately identified, and that they could attend to and properly decode social cues. However, future studies might consider adding participants with a formal diagnosis of autism to compare against low and high autistic trait individuals. Another potential limitation might be the participants, themselves. As they were all university students, one could argue that admission into university implies a higher level of functioning, and thus the performance of high autistic trait individuals would be expected to mimic their peers lower in autistic traits. Future studies should consider diversifying the sample. Nevertheless, to our knowledge, this is the first study to examine the ability of high autistic trait individuals to assess the group status of others in a naturalistic, albeit video-based [102], setting; and it is the first to investigate the way that they assess others (e.g., prestige vs. dominance) in attaining that status. With a larger sample size, a future study could probe further and examine if HAQ equate status with dominance or prestige, and whether or not that differs from LAQ.

## Conclusion

In sum, the current study examined the gaze behaviour of individuals from the general population who are high in autistic traits while viewing dynamic social interactions, and their ability to assess others’ social standing within a group. The results indicate that the individuals who scored high on the AQ-10, and individuals who did not, performed similarly in terms of gaze behaviour and peer assessment. In short, high autistic trait individuals are remarkably similar to low autistic trait individuals in how and where they look during social interactions, and in how they interpret their observations. Although this was not predicted, it is perhaps an indication that university-aged individuals high in autistic traits have the ability to “read the room”.

## References

[1] American Psychiatric Association, *Diagnostic and statistical manual of mental disorders*: *DSM-5*^™^, 5th ed. American Psychiatric Publishing, Inc., 2013.

[2] M. Ofner et al., “Autism spectrum disorder among children and youth in Canada 2018: A report of the National Autism Spectrum Disorder Surveillance System,” 2018. [Online]. https://www.canada.ca/en/public-health/services/publications/diseases-conditions/autism-spectrum-disorder-children-youth-canada-2018.html. [Accessed: 17-Jan-2020].

[3] Carpenter L. A., Soorya L., and Halpern D., “Asperger ‘ s Syndrome and High-Functioning Autism,” no. January, pp. 30–36, 2009. 10.3928/00904481-20090101-01 19213291

[4] Shriberg L. D., Paul R., McSweeny J. L., Klin A., Cohen D. J., and Volkmar F. R., “Speech and prosody characteristics of adolescents and adults with high-functioning autism and Asperger syndrome,” *J*. *Speech*, *Lang*. *Hear*. *Res*., vol. 44, no. 5, pp. 1097–1115, 2001. doi: 10.1044/1092-4388(2001/087) 11708530

[5] Bauminger N. and Kasari C., “Loneliness and friendship in high-functioning children with autism.,” *Child Dev*., vol. 71, no. 2, pp. 447–456, 2000. doi: 10.1111/1467-8624.00156 10834476

[6] Gutstein S. E. and Whitney T., “Asperger Syndrome and the Development of Social Competence,” *Focus Autism Other Dev*. *Disabl*., vol. 17, no. 3, pp. 161–171, 2002.

[7] Constantino J. N. and Todd R. D., “Autistic Traits in the General Population,” *Arch*. *Gen*. *Psychiatry*, vol. 60, no. 5, p. 524, May 2003. 12742874 10.1001/archpsyc.60.5.524

[8] Baron-Cohen S., Wheelwright S., Hill J., Raste Y., and Plumb I., “The ‘Reading the Mind in the Eyes’ Test revised version: a study with normal adults, and adults with Asperger syndrome or high-functioning autism.,” *J*. *Child Psychol*. *Psychiatry*., vol. 42, no. 2, pp. 241–51, Feb. 2001. 11280420

[9] Constantino J. N. et al., “Autistic Social Impairment in the Siblings of Children With Pervasive Developmental Disorders,” *Am*. *J*. *Psychiatry*, vol. 163, no. 2, pp. 294–296, Feb. 2006. doi: 10.1176/appi.ajp.163.2.294 16449484

[10] Bailey A. et al., “Autism as a strongly genetic disorder: evidence from a British twin study,” *Psychol*. *Med*., vol. 25, no. 1, pp. 63–77, Jan. 1995. doi: 10.1017/s0033291700028099 7792363

[11] Cha W.-J. and Lee J.-H., “Diminished ability to integrate target stimuli with context during emotional recognition in individuals with broad autism phenotype,” *Front*. *Psychol*., vol. 13, Oct. 2022. doi: 10.3389/fpsyg.2022.934385 36275254 PMC9583922

[12] De Groot K. and Van Strien J. W., “Evidence for a Broad Autism Phenotype,” *Adv*. *Neurodev*. *Disord*., vol. 1, no. 3, pp. 129–140, Sep. 2017.

[13] Morrison K. E., Chambers L. K., Faso D. J., and Sasson N. J., “The content and function of interests in the broad autism phenotype,” *Res*. *Autism Spectr*. *Disord*., vol. 49, pp. 25–33, May 2018.

[14] Sasson N. J., Nowlin R. B., and Pinkham A. E., “Social cognition, social skill, and the broad autism phenotype,” *Autism*, vol. 17, no. 6, pp. 655–667, Nov. 2013. doi: 10.1177/1362361312455704 22987889

[15] St. Pourcain B. et al., “Association Between a High-Risk Autism Locus on 5p14 and Social Communication Spectrum Phenotypes in the General Population,” *Am*. *J*. *Psychiatry*, vol. 167, no. 11, pp. 1364–1372, Nov. 2010. doi: 10.1176/appi.ajp.2010.09121789 20634369 PMC3008767

[16] Howlin P., Moss P., Savage S., Bolton P., and Rutter M., “Outcomes in Adult Life Among Siblings of Individuals with Autism,” *J*. *Autism Dev*. *Disord*., vol. 45, no. 3, pp. 707–718, Mar. 2015. doi: 10.1007/s10803-014-2224-5 25189825

[17] Gerdts J. and Bernier R., “The Broader Autism Phenotype and Its Implications on the Etiology and Treatment of Autism Spectrum Disorders,” *Autism Res*. *Treat*., vol. 2011, pp. 1–19, 2011. doi: 10.1155/2011/545901 22937250 PMC3420416

[18] Sucksmith E., Roth I., and Hoekstra R. A., “Autistic Traits Below the Clinical Threshold: Re-examining the Broader Autism Phenotype in the 21st Century,” *Neuropsychol*. *Rev*., vol. 21, no. 4, pp. 360–389, Dec. 2011. doi: 10.1007/s11065-011-9183-9 21989834

[19] Briskman J., Happe F., and Frith U., “Exploring the Cognitive Phenotype of Autism: Weak ‘Central Coherence’ in Parents and Siblings of Children with Autism: II. Real-life Skills and Preferences,” *J*. *Child Psychol*. *Psychiatry*, vol. 42, no. 3, pp. 309–316, Mar. 2001. 11321200

[20] Poljac E., Poljac E., and Wagemans J., “Reduced accuracy and sensitivity in the perception of emotional facial expressions in individuals with high autism spectrum traits,” *Autism*, vol. 17, no. 6, pp. 668–680, Nov. 2013. doi: 10.1177/1362361312455703 22987888

[21] Bernier R., Gerdts J., Munson J., Dawson G., and Estes A., “Evidence for broader autism phenotype characteristics in parents from multiple-incidence autism families,” *Autism Res*., vol. 5, no. 1, pp. 13–20, Feb. 2012. doi: 10.1002/aur.226 21905246 PMC3237782

[22] Ruser T. F. et al., “Communicative competence in parents of children with autism and parents of children with specific language impairment,” *J*. *Autism Dev*. *Disord*., vol. 37, no. 7, pp. 1323–1336, 2007. doi: 10.1007/s10803-006-0274-z 17180460

[23] Bolton P. et al., “A Case‐Control Family History Study of Autism,” *J*. *Child Psychol*. *Psychiatry*, vol. 35, no. 5, pp. 877–900, 1994. doi: 10.1111/j.1469-7610.1994.tb02300.x 7962246

[24] Camodeca A., “Real-World Executive Functioning and Subclinical Autism Traits in Autism Parents, Other Disability Parents, and Non-Clinical Undergraduates,” *Curr*. *Psychol*., Jan. 2022.

[25] Landry O. and Chouinard P. A., “Why We Should Study the Broader Autism Phenotype in Typically Developing Populations,” *J*. *Cogn*. *Dev*., vol. 17, no. 4, pp. 584–595, Aug. 2016.

[26] Dwyer P., Ryan J. G., Williams Z. J., and Gassner D. L., “First Do No Harm: Suggestions Regarding Respectful Autism Language,” *Pediatrics*, vol. 149, no. Supplement 4, Apr. 2022. doi: 10.1542/peds.2020-049437N 35363298 PMC9066426

[27] Baron-Cohen S., Leslie A. M., and Frith U., “Does the autistic child have a ‘theory of mind’?,” *Cognition*, vol. 21, no. 1, pp. 37–46, Oct. 1985. 2934210 10.1016/0010-0277(85)90022-8

[28] Andreou M. and Skrimpa V., “Theory of Mind Deficits and Neurophysiological Operations in Autism Spectrum Disorders: A Review,” *Brain Sci*., vol. 10, no. 6, p. 393, Jun. 2020. doi: 10.3390/brainsci10060393 32575672 PMC7349236

[29] Bowler D. M., “‘Theory of Mind’ in Asperger’s Syndrome Dermot M. Bowler,” *J*. *Child Psychol*. *Psychiatry*, vol. 33, no. 5, pp. 877–893, Jul. 1992. 1378848 10.1111/j.1469-7610.1992.tb01962.x

[30] Happé F., “An advanced test of theory of mind: Understanding of story characters’ thoughts and feelings by able autistic, mentally handicapped, and normal children and adults,” *J*. *Autism Dev*. *Disord*., vol. 24, no. 2, pp. 129–154, 1994. doi: 10.1007/BF02172093 8040158

[31] Rajendran G. and Mitchell P., “Cognitive theories of autism,” *Dev*. *Rev*., vol. 27, no. 2, pp. 224–260, Jun. 2007.

[32] Eyuboglu M., Baykara B., and Eyuboglu D., “Broad autism phenotype: theory of mind and empathy skills in unaffected siblings of children with autism spectrum disorder,” *Psychiatry Clin*. *Psychopharmacol*., vol. 28, no. 1, pp. 36–42, Jan. 2018.

[33] Atherton G. and Cross L., “Animal Faux Pas: Two Legs Good Four Legs Bad for Theory of Mind, but Not in the Broad Autism Spectrum,” *J*. *Genet*. *Psychol*., vol. 180, no. 2–3, pp. 81–95, May 2019. doi: 10.1080/00221325.2019.1593100 31094293

[34] Happé F. and Frith U., “The weak coherence account: Detail-focused cognitive style in autism spectrum disorders,” *J*. *Autism Dev*. *Disord*., vol. 36, no. 1, pp. 5–25, 2006. doi: 10.1007/s10803-005-0039-0 16450045

[35] López B., Donnelly N., Hadwin J. A., and Leekam S. R., “Face processing in high-functioning adolescents with autism: Evidence for weak central coherence,” *Vis*. *cogn*., vol. 11, no. 6, pp. 673–688, 2004.

[36] Morgan B., Maybery M., and Durkin K., “Weak Central Coherence, Poor Joint Attention, and Low Verbal Ability: Independent Deficits in Early Autism,” *Dev*. *Psychol*., vol. 39, no. 4, pp. 646–656, 2003. doi: 10.1037/0012-1649.39.4.646 12859119

[37] Chevallier C., Kohls G., Troiani V., Brodkin E. S., and Schultz R. T., “The social motivation theory of autism,” *Trends in Cognitive Sciences*. 2012. doi: 10.1016/j.tics.2012.02.007 22425667 PMC3329932

[38] Kohls G. et al., “Atypical brain responses to reward cues in autism as revealed by event-related potentials,” *J*. *Autism Dev*. *Disord*., vol. 41, no. 11, pp. 1523–1533, 2011. doi: 10.1007/s10803-011-1177-1 21290174

[39] Clements C. C., Zoltowski A. R., Yankowitz L. D., Yerys B. E., Schultz R. T., and Herrington J. D., “Evaluation of the social motivation hypothesis of autism a systematic review and meta-analysis,” *JAMA Psychiatry*, vol. 75, no. 8, pp. 797–808, Aug. 2018. doi: 10.1001/jamapsychiatry.2018.1100 29898209 PMC6143096

[40] Scott-Van Zeeland A. A., Dapretto M., Ghahremani D. G., Poldrack R. A., and Bookheimer S. Y., “Reward processing in autism,” *Autism Res*., vol. 3, no. 2, pp. 53–67, 2010. doi: 10.1002/aur.122 20437601 PMC3076289

[41] Dichter G. S., Richey J. A., Rittenberg A. M., Sabatino A., and Bodfish J. W., “Reward circuitry function in autism during face anticipation and outcomes,” *J*. *Autism Dev*. *Disord*., vol. 42, no. 2, pp. 147–160, 2012. doi: 10.1007/s10803-011-1221-1 22187105 PMC8624275

[42] Cox A. et al., “Diminished social reward anticipation in the broad autism phenotype as revealed by event-related brain potentials,” *Soc*. *Cogn*. *Affect*. *Neurosci*., vol. 10, no. 10, pp. 1357–1364, Oct. 2015. doi: 10.1093/scan/nsv024 25752905 PMC4590535

[43] Tottenham N., Hertzig M. E., Gillespie-Lynch K., Gilhooly T., Millner A. J., and Casey B. J., “Elevated amygdala response to faces and gaze aversion in autism spectrum disorder,” *Soc*. *Cogn*. *Affect*. *Neurosci*., vol. 9, no. 1, pp. 106–117, 2014. doi: 10.1093/scan/nst050 23596190 PMC3871735

[44] Tanaka J. W. and Sung A., “The ‘Eye Avoidance’ Hypothesis of Autism Face Processing,” *J*. *Autism Dev*. *Disord*., vol. 46, no. 5, 2016. doi: 10.1007/s10803-013-1976-7 24150885 PMC3997654

[45] Senju A. and Johnson M. H., “Atypical eye contact in autism: Models, mechanisms and development,” *Neurosci*. *Biobehav*. *Rev*., vol. 33, no. 8, pp. 1204–1214, 2009. doi: 10.1016/j.neubiorev.2009.06.001 19538990

[46] Modahl C. et al., “Plasma oxytocin levels in autistic children,” *Biol*. *Psychiatry*, vol. 43, no. 4, pp. 270–277, 1998. doi: 10.1016/s0006-3223(97)00439-3 9513736

[47] Bartz J. A., Zaki J., Bolger N., and Ochsner K. N., “Social effects of oxytocin in humans: Context and person matter,” *Trends Cogn*. *Sci*., vol. 15, no. 7, pp. 301–309, 2011. doi: 10.1016/j.tics.2011.05.002 21696997

[48] Pieslinger J., Wiskerke J., and Igelström K. M., “Stratifying the broad autistic phenotype: Contributions of social anhedonia, mentalizing and face blindness to social autistic traits.” 2022. 10.3389/fnbeh.2022.1046097 PMC9817135 36620857

[49] Carré A. et al., “Tracking Social Motivation Systems Deficits: The Affective Neuroscience View of Autism,” *J*. *Autism Dev*. *Disord*., vol. 45, no. 10, pp. 3351–3363, Oct. 2015. doi: 10.1007/s10803-015-2498-2 26123007

[50] Nayar K., Shic F., Winston M., and Losh M., “A constellation of eye-tracking measures reveals social attention differences in ASD and the broad autism phenotype,” *Mol*. *Autism*, vol. 13, no. 1, p. 18, Dec. 2022. doi: 10.1186/s13229-022-00490-w 35509089 PMC9069739

[51] Dalton K. M., Nacewicz B. M., Alexander A. L., and Davidson R. J., “Gaze-Fixation, Brain Activation, and Amygdala Volume in Unaffected Siblings of Individuals with Autism,” *Biol*. *Psychiatry*, vol. 61, no. 4, pp. 512–520, Feb. 2007. doi: 10.1016/j.biopsych.2006.05.019 17069771

[52] Guastella A. J. et al., “Intranasal Oxytocin Improves Emotion Recognition for Youth with Autism Spectrum Disorders,” *Biol*. *Psychiatry*, vol. 67, no. 7, pp. 692–694, Apr. 2010. doi: 10.1016/j.biopsych.2009.09.020 19897177

[53] Mu E., “Intranasal oxytocin normalises early visual evoked potentials to emotional faces for individuals with high autistic traits,” Swinburne University of Technology, 2021.

[54] Ristic J., Mottron L., Friesen C. K., Iarocci G., Burack J. A., and Kingstone A., “Eyes are special but not for everyone: The case of autism,” *Cogn*. *Brain Res*., vol. 24, no. 3, pp. 715–718, 2005. 10.1016/j.cogbrainres.2005.02.007 16099372

[55] Adler N., Nadler B., Eviatar Z., and Shamay-Tsoory S. G., “The relationship between theory of mind and autobiographical memory in high-functioning autism and Asperger syndrome,” *Psychiatry Res*., vol. 178, no. 1, pp. 214–216, 2010. doi: 10.1016/j.psychres.2009.11.015 20452047

[56] Peterson C. C., Slaughter V., and Brownell C., “Children with autism spectrum disorder are skilled at reading emotion body language,” *J*. *Exp*. *Child Psychol*., 2015. doi: 10.1016/j.jecp.2015.04.012 26079273

[57] Montgomery C. B., Allison C., Lai M. C., Cassidy S., Langdon P. E., and Baron-Cohen S., “Do Adults with High Functioning Autism or Asperger Syndrome Differ in Empathy and Emotion Recognition?,” *J*. *Autism Dev*. *Disord*., vol. 46, no. 6, pp. 1931–1940, 2016. doi: 10.1007/s10803-016-2698-4 26883645 PMC4860194

[58] Pazhoohi F., Forby L., and Kingstone A., “Facial masks affect emotion recognition in the general population and individuals with autistic traits,” *PLoS One*, vol. 16, no. 9, p. e0257740, Sep. 2021. doi: 10.1371/journal.pone.0257740 34591895 PMC8483373

[59] Elder L. M., Caterino L. C., Janet C., Shacknai D., and De Simone G., “The efficacy of social skills treatment for children with Asperger Syndrome,” *Educ*. *Treat*. *Child*., vol. 29, no. 4, pp. 635–663, 2006.

[60] Laugeson E. A. and Ellingsen R., “Social Skills Training for Adolescents and Adults with Autism Spectrum Disorder,” in *Adolescents and Adults with Autism Spectrum Disorders*, New York, NY: Springer New York, 2014, pp. 61–85.

[61] Fast Y., *Employment for individuals with Asperger syndrome or non-verbal learning disability*: *Stories and strategies*. Jessica Kingsley Publishers, 2004.

[62] Telzrow C. F. and Koch L. C., “Nonverbal Learning Disability: Vocational Implications and Rehabilitation Treatment Approaches,” *J*. *Appl*. *Rehabil*. *Couns*., vol. 34, no. 2, pp. 9–16, 2003.

[63] Higgins K. K., Koch L. C., Boughfman E. M., and Vierstra C., “School-to-work transition and asperger syndrome,” *Work*, vol. 31, no. 3, pp. 291–298, 2008. 19029670

[64] Gil-White F. J. and Henrich J., “The Evolution of Prestige,” *Evolution and Human Behavior*, vol. 22, no. 2. pp. 165–196, 2001. 11384884 10.1016/s1090-5138(00)00071-4

[65] Cheng J. T., Tracy J. L., Foulsham T., Kingstone A., and Henrich J., “Two ways to the top: Evidence that dominance and prestige are distinct yet viable avenues to social rank and influence.,” *J*. *Pers*. *Soc*. *Psychol*., vol. 104, no. 1, pp. 103–125, 2013. doi: 10.1037/a0030398 23163747

[66] Maner J. K., “Dominance and Prestige: A Tale of Two Hierarchies,” *Curr*. *Dir*. *Psychol*. *Sci*., vol. 26, no. 6, pp. 526–531, Dec. 2017.

[67] Cheng J. T., “Dominance, prestige, and the role of leveling in human social hierarchy and equality,” *Curr*. *Opin*. *Psychol*., vol. 33, pp. 238–244, Jun. 2020. doi: 10.1016/j.copsyc.2019.10.004 31794955

[68] de Waal-Andrews W., Gregg A. P., and Lammers J., “When status is grabbed and when status is granted: Getting ahead in dominance and prestige hierarchies,” *Br*. *J*. *Soc*. *Psychol*., vol. 54, no. 3, pp. 445–464, Sep. 2015. doi: 10.1111/bjso.12093 25370539

[69] Henrich J., Chudek M., and Boyd R., “The big man mechanism: How prestige fosters cooperation and creates Prosocial leaders,” *Philos*. *Trans*. *R*. *Soc*. *B Biol*. *Sci*., vol. 370, no. 1683, 2015. 10.1098/rstb.2015.0013 PMC4633849 26503686

[70] Weidman A. C., Cheng J. T., and Tracy J. L., “The psychological structure of humility,” *J*. *Pers*. *Soc*. *Psychol*., vol. 114, no. 1, pp. 153–178, 2018. doi: 10.1037/pspp0000112 27454926

[71] Foulsham T., Cheng J. T., Tracy J. L., Henrich J., and Kingstone A., “Gaze allocation in a dynamic situation: Effects of social status and speaking,” *Cognition*, vol. 117, no. 3, pp. 319–331, Dec. 2010. doi: 10.1016/j.cognition.2010.09.003 20965502

[72] Allison C., Auyeung B., and Baron-Cohen S., “Toward brief ‘red flags’ for autism screening: The short Autism Spectrum Quotient and the short Quantitative Checklist in 1,000 cases and 3,000 controls,” *J*. *Am*. *Acad*. *Child Adolesc*. *Psychiatry*, vol. 51, no. 2, pp. 202–212.e7, 2012. 22265366 10.1016/j.jaac.2011.11.003

[73] Rhind C. et al., “An examination of autism spectrum traits in adolescents with anorexia nervosa and their parents,” *Mol*. *Autism*, vol. 5, no. 1, pp. 1–9, 2014. 25553237 10.1186/2040-2392-5-56 PMC4280745

[74] Tchanturia K. et al., “Exploring autistic traits in anorexia: A clinical study,” *Mol*. *Autism*, vol. 4, no. 1, p. 1, 2013. 24220604 10.1186/2040-2392-4-44 PMC4176300

[75] Kristensen Z. E. and Broome M. R., “Autistic Traits in an Internet Sample of Gender Variant UK Adults,” *Int*. *J*. *Transgenderism*, vol. 16, no. 4, pp. 234–245, 2015.

[76] Stanyon D. et al., “The role of bullying victimization in the pathway between autistic traits and psychotic experiences in adolescence: Data from the Tokyo Teen Cohort study,” *Schizophr*. *Res*., vol. 239, no. December 2021, pp. 111–115, 2022. doi: 10.1016/j.schres.2021.11.015 34871995

[77] Unruh K. E. et al., “Social orienting and attention is influenced by the presence of competing nonsocial information in adolescents with autism,” *Front*. *Neurosci*., vol. 10, no. DEC, 2016. doi: 10.3389/fnins.2016.00586 28066169 PMC5179566

[78] Dubey I., Ropar D., and Hamilton A. F., “Measuring the value of social engagement in adults with and without autism,” *Mol*. *Autism*, vol. 6, no. 1, pp. 1–9, 2015. doi: 10.1186/s13229-015-0031-2 26097674 PMC4473830

[79] Sasson N. J., Dichter G. S., and Bodfish J. W., “Affective responses by adults with autism are reduced to social images but elevated to images related to circumscribed interests,” *PLoS One*, vol. 7, no. 8, 2012. doi: 10.1371/journal.pone.0042457 22870328 PMC3411654

[80] Watson K. K. et al., “Increased reward value of non-social stimuli in children and adolescents with autism,” *Front*. *Psychol*., vol. 6, no. July, pp. 1–8, 2015. doi: 10.3389/fpsyg.2015.01026 26257684 PMC4510834

[81] Osterling J. A. and Dawson G., “Early recognition of children with autism: A study of first birthday home videotapes,” *J*. *Autism Dev*. *Disord*., vol. 24, no. 3, pp. 247–257, 1994. doi: 10.1007/BF02172225 8050980

[82] Osterling J. A., Dawson G., and Munson J. A., “Early recognition of 1-year-old infants with autism spectrum disorder versus mental retardation,” *Dev*. *Psychopathol*., vol. 14, no. 2, pp. 239–251, 2002. doi: 10.1017/s0954579402002031 12030690

[83] Dapretto M. et al., “Understanding emotions in others: Mirror neuron dysfunction in children with autism spectrum disorders,” *Nat*. *Neurosci*., vol. 9, no. 1, pp. 28–30, 2006. doi: 10.1038/nn1611 16327784 PMC3713227

[84] Rutherford M. D. and Towns A. M., “Scan path differences and similarities during emotion perception in those with and without autism spectrum disorders,” *J*. *Autism Dev*. *Disord*., vol. 38, no. 7, pp. 1371–1381, 2008. doi: 10.1007/s10803-007-0525-7 18297386

[85] Biesanz J., “Fabs: Functions for Applied Behavioural Sciences.” 2018.

[86] R Core Team, “R: A language and environment for statistical computing,” 2018.

[87] Bottger P. C., “Expertise and air time as bases of actual and perceived influence in problem-solving groups.,” *J*. *Appl*. *Psychol*., vol. 69, no. 2, pp. 214–221, 1984.

[88] Cheng J. T., Tracy J. L., and Henrich J., “Pride, personality, and the evolutionary foundations of human social status,” *Evol*. *Hum*. *Behav*., vol. 31, no. 5, pp. 334–347, Sep. 2010.

[89] Booth T., Murray A. L., McKenzie K., Kuenssberg R., O’Donnell M., and Burnett H., “Brief report: An evaluation of the AQ-10 as a brief screening instrument for asd in adults,” *J*. *Autism Dev*. *Disord*., vol. 43, no. 12, pp. 2997–3000, 2013. doi: 10.1007/s10803-013-1844-5 23640304

[90] H. Singmann, B. Boker, J. Westfall, F. Aust, and M. S. Ben-Shachar, “afex: Analysis of Factorial Experiments.” https://CRAN.R-project.org/package=afex, R package version 0.28–1, 2021.

[91] A. Kassambara, “rstatix: Pipe-Friendly Framework for Basic Statistical Tests.” https://CRAN.R-project.org/package=rstatix, R package version 0.7.0., 2021.

[92] R. V. Lenth, “emmeans: Estimated Marginal Means, aka Least-Squares Means.” https://CRAN.R-project.org/package=emmeans, R package version 1.6, 2021.

[93] Wickham H., “ggplot2: Elegant Graphics for Data Analysis.” Springer-Verlag New York, 2016.

[94] Happe F., “Autism: cognitive deficit or cognitive style?,” *Trends Cogn*. *Sci*., vol. 3, no. 6, pp. 216–222, 1999. doi: 10.1016/s1364-6613(99)01318-2 10354574

[95] Ogawa S., Iriguchi M., Lee Y. A., Yoshikawa S., and Goto Y., “Atypical Social Rank Recognition in Autism Spectrum Disorder,” *Sci*. *Rep*., vol. 9, no. 1, pp. 1–6, 2019. 31666630 10.1038/s41598-019-52211-8 PMC6821924

[96] Howlin P., “Outcome in adult life for more able individuals with autism or Asperger syndrome,” *Autism*, vol. 4, no. 1, pp. 63–83, 2000.

[97] Humphrey N. and Symes W., “Peer interaction patterns among adolescents with autistic spectrum disorders (ASDs) in mainstream school settings,” *Autism*, vol. 15, no. 4, pp. 397–419, 2011. 21454385 10.1177/1362361310387804

[98] Trevisan D., Roberts N., Lin C. J., and Birmingham E., “How do individuals with Autism experience eye contact? A qualitative analysis of first-hand accounts,” *PLoS One*, vol. in revisio, pp. 1–22, 2017. 10.1371/journal.pone.0188446 PMC5705114 29182643

[99] Hadjikhani N. et al., “Bumetanide for autism: More eye contact, less amygdala activation,” *Sci*. *Rep*., vol. 8, no. 1, pp. 8–15, 2018. 29483603 10.1038/s41598-018-21958-x PMC5827728

[100] Clutterbuck R. A., Shah P., Leung H. S., Callan M. J., Gjersoe N., and Livingston L. A., “Anthropomorphic tendencies in autism: A conceptual replication and extension of White and Remington (2019) and preliminary development of a novel anthropomorphism measure,” *Autism*, vol. 26, no. 4, pp. 940–950, May 2022. doi: 10.1177/13623613211039387 34538099 PMC9014771

[101] White R. C. and Remington A., “Object personification in autism: This paper will be very sad if you don’t read it,” *Autism*, vol. 23, no. 4, pp. 1042–1045, May 2019. doi: 10.1177/1362361318793408 30101594

[102] Risko E. F., Laidlaw K., Freeth M., Foulsham T., and Kingstone A., “Social attention with real versus reel stimuli: toward an empirical approach to concerns about ecological validity,” *Front*. *Hum*. *Neurosci*., vol. 6, 2012. doi: 10.3389/fnhum.2012.00143 22654747 PMC3360477

